# Targeting the Odontogenic Keratocysts: Therapeutic Potential of 5-Fluorouracil Through Thymidylate Synthase Inhibition

**DOI:** 10.7759/cureus.82905

**Published:** 2025-04-24

**Authors:** G V Reddy, G. Siva Prasada Reddy, Vishnu Priyatham, John Jims Veeravalli, Srimounica Kocherlakota

**Affiliations:** 1 Department of Oral and Maxillofacial Surgery, Panineeya Mahavidyalaya Institute of Dental Sciences and Research Centre, Hyderabad, IND

**Keywords:** 5-fluorouracil, odontogenic keratocyst, recurrence, targeted therapy, thymidylate synthase inhibition

## Abstract

Odontogenic keratocysts (OKCs) are aggressive cystic lesions of the jaw with a high recurrence rate. They are benign lesions that arise from dental lamina remnants or basal cells of the overlying epithelium. Various surgical and adjunctive treatments have been explored to minimize recurrence. In this report, we review the literature elucidating the efficacy of 5-fluorouracil (5-FU) topical application for recurrent OKCs and discuss the management of an OKC with 5-FU after enucleation and a 12-month follow-up.

## Introduction

Odontogenic keratocysts (OKCs) are unique and clinically significant lesions due to their aggressive nature, high recurrence rate, and ongoing debates regarding optimal treatment strategies. Phillipsen and Reichart initially identified the OKC as a distinct pathological entity in 1956 [1]. However, the World Health Organization (WHO) reclassified it as a keratocystic odontogenic tumor (KCOT) in 2005 as a result of its high recurrence potential and association with genetic syndromes, including Gorlin-Goltz syndrome [2]. Nevertheless, the 2017 WHO classification reverted to the term OKC, citing a lack of evidence to substantiate its neoplastic nature [2].

The mandible, notably the posterior region, is the primary site of OKCs, which are derived from the dental lamina [3]. They frequently remain asymptomatic until they experience a substantial increase in size, which typically affects the mandibular ramus and body. Radiographically, they are observed as unilocular or multilocular radiolucencies, such as ameloblastomas or dentigerous cysts [4].

Parakeratotic and orthokeratotic subtypes are the two categories into which OKCs are classified histopathologically. The parakeratotic variant is distinguished by an increase in keratin production, the absence of keratolytic granules, and the sloughing of epithelial cells into the keratin layer, while the orthokeratotic variant displays distinct keratinization patterns [5]. Insight into the biological behavior of OKCs is provided by this classification. A comprehensive clinical evaluation, radiographic imaging, and histopathological confirmation are necessary for the diagnosis. The treatment approach for OKCs remains contentious, with recurrence rates spanning from less than 10% to over 60% [6]. Common treatment modalities include enucleation, decompression, marsupialization, and enucleation with adjunctive techniques, including liquid nitrogen cryotherapy, peripheral ostectomy, chemical curettage using Carnoy's solution (CS), or, in severe cases, mandible resection. Despite the fact that CS was once the preferred option due to its ability to reduce recurrence, its use has decreased as a result of the FDA's prohibition of chloroform, a key ingredient with carcinogenic potential. Modified Carnoy’s solution (MCS), which lacks chloroform, is now used but is considered less effective [7].

Several theories explain OKC recurrence, related to incomplete removal of the cyst lining, growth from satellite cysts, persistence of odontogenic epithelial remnants, and the development of new OKCs in adjacent areas. Higher recurrence rates are attributed to factors such as infection, fistula formation, bony wall perforation, and multilocular OKCs being particularly susceptible to recurrence [8]. Furthermore, recurrence tendencies are influenced by molecular characteristics and proliferative markers [9].

A potential adjunctive therapy for the management of OKCs has been identified as a novel approach that involves the topical application of 5-fluorouracil (5-FU). It acts by inhibiting thymidylate synthetase (TS), an enzyme essential for DNA synthesis. This antimetabolite, which is frequently employed to treat basal cell carcinoma (BCC) and actinic keratosis, targets proliferating cells, resulting in apoptosis of tumor cells [10]. Molecular-targeted therapies have been investigated because OKCs, including BCC, are linked to mutations in the PTCH1 gene and the activation of the Sonic Hedgehog (SHH) pathway. There is evidence to suggest that the incidence and morbidity of OKCs in patients with nevoid basal cell carcinoma syndrome can be reduced by inhibiting the SHH pathway with agents like vismodegib [11].

Compared to traditional adjunctive therapies such as MCS and liquid nitrogen, 5-FU offers a more targeted antiproliferative approach. It is applied topically on ribbon gauze placed within the bone cavity post-curettage and peripheral ostectomy, ensuring direct exposure of residual OKC epithelium to the drug. Unlike CS, which requires meticulous precautions to prevent damage to adjacent tissues, 5-FU application is simpler and does not pose neurotoxic risks. However, thorough application is critical, as areas lacking contact with 5-FU-coated gauze may lead to recurrence.

Given the molecular similarities between OKCs and BCC, the integration of targeted molecular therapies such as 5-FU and SHH pathway inhibitors could revolutionize OKC management. This case highlights the potential of 5-FU as an effective adjunct in reducing recurrence. Further clinical studies are warranted to validate its efficacy and establish standardized treatment protocols for OKCs.

## Case presentation

A 35-year-old female patient presented to the department with a chief complaint of pain and swelling on the right side of the face persisting for one month. Her past dental and medical history was non-contributory.

**Figure 1:**
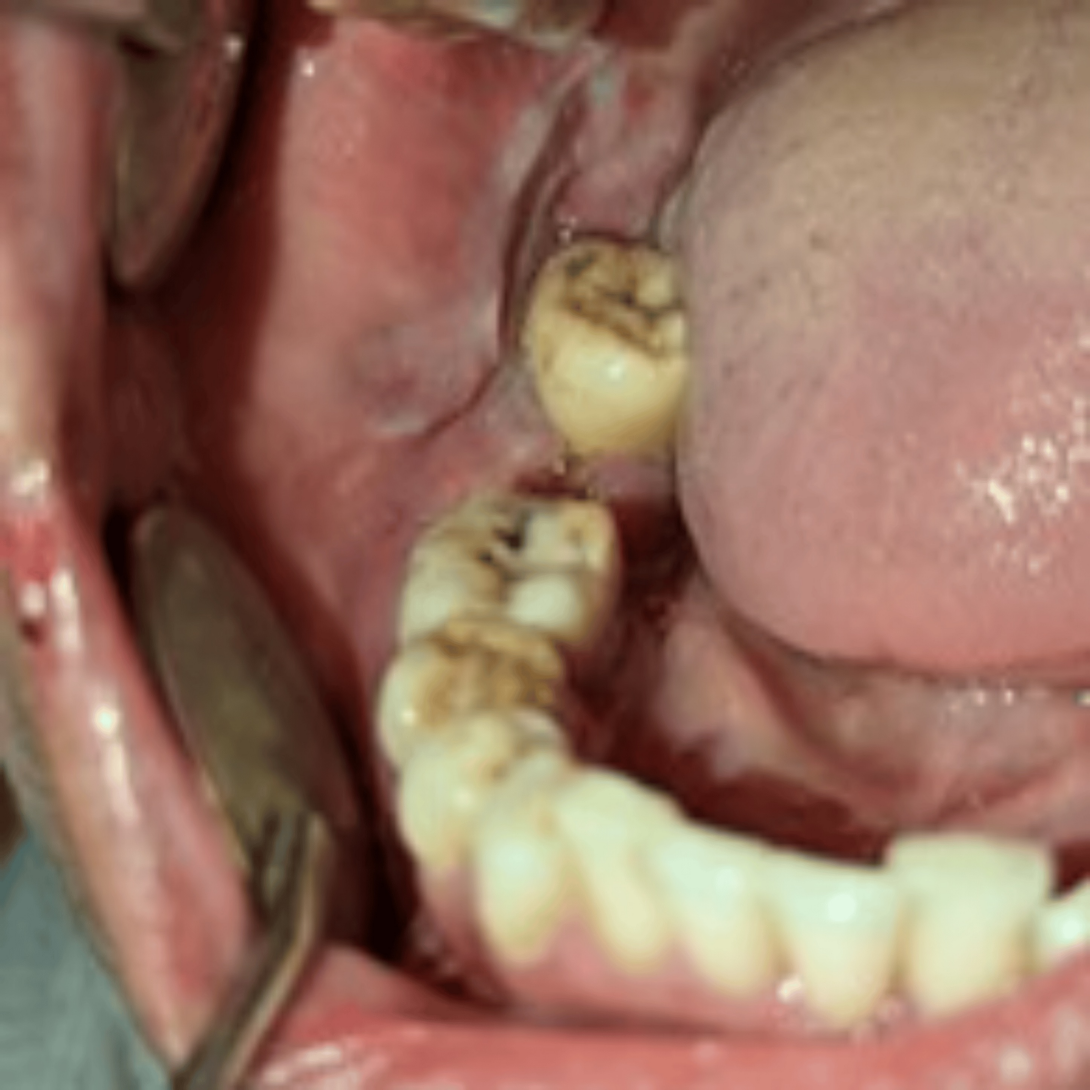
Intraoral examination revealed a diffuse swelling on the right buccal mucosa, with obliteration of the vestibule in the region of teeth #46, #47, and #48

Extraoral examination revealed gross facial asymmetry with a swelling involving the right middle and lower third of the face. Tenderness was noted on palpation in the right-angle region of the mandible. No signs of paresthesia were present. A single, palpable right submandibular lymph node was identified, which was firm, tender, and mobile. Intraoral examination revealed a diffuse swelling on the right buccal mucosa, with obliteration of the vestibule in the region of teeth #46, #47, and #48 (Figure 1).

**Figure 2:**
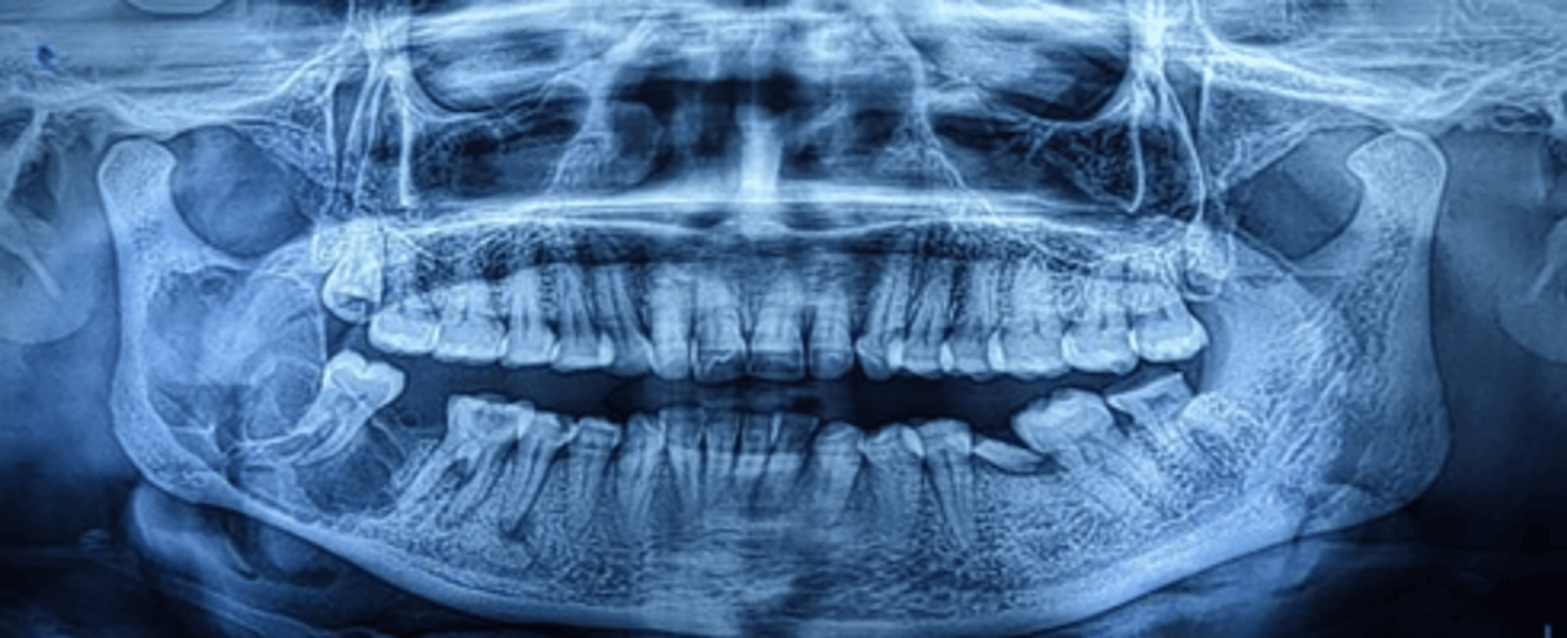
Orthopantomogram (OPG) revealed a well-defined multilocular radiolucency with curved septa involving the right body and ramus of the mandible

A preoperative orthopantomogram (OPG) revealed a well-defined multilocular radiolucency with curved septa involving the right body and ramus of the mandible (Figure 2).

**Figure 3:**
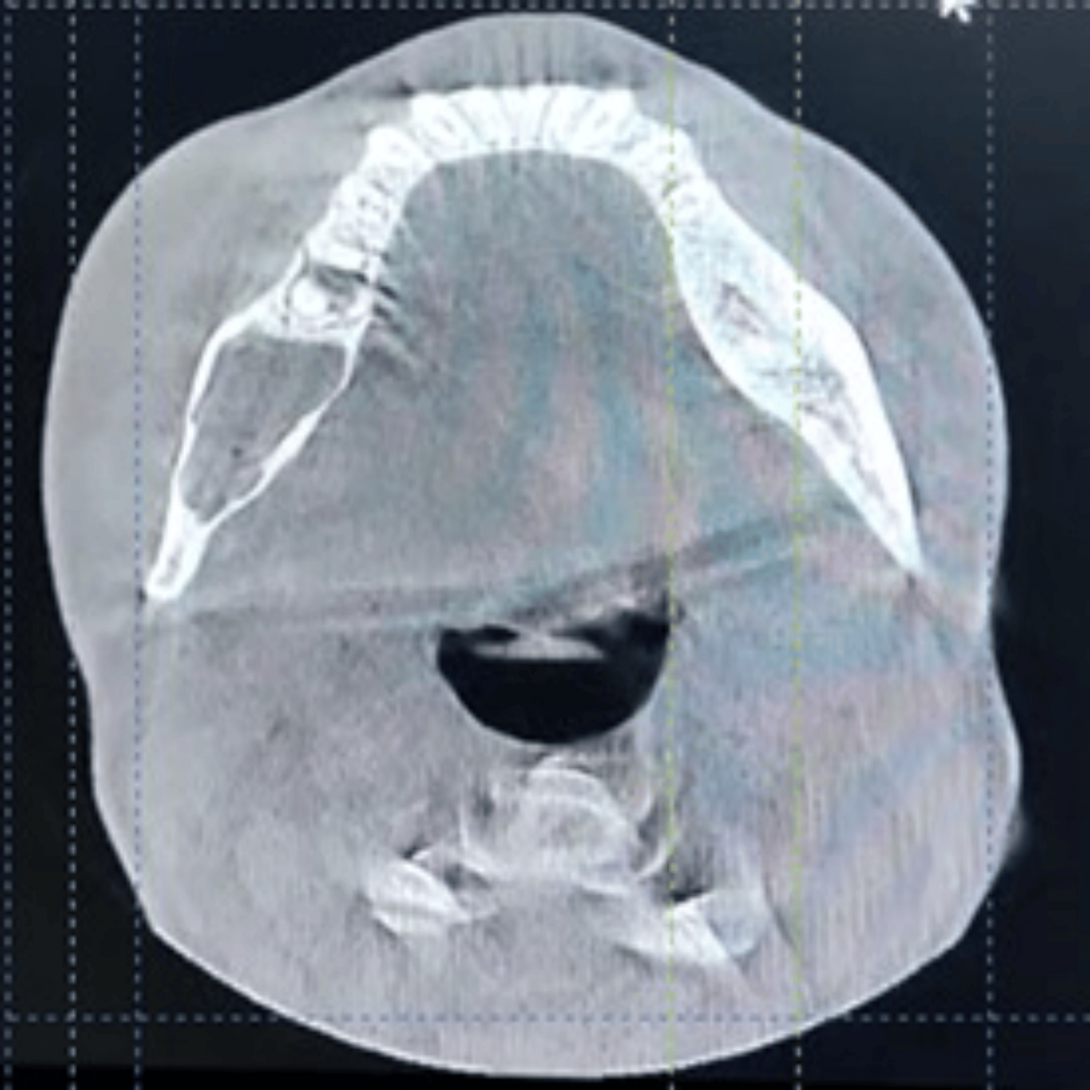
Computed tomography (CT) demonstrated expansion and thinning of the buccal and lingual cortical plates, with thinning and perforation of the medial surface of the ramus of the mandible

Computed tomography (CT) demonstrated expansion and thinning of the buccal and lingual cortical plates, with thinning and perforation of the medial surface of the ramus of the mandible (Figure 3).

**Figure 4:**
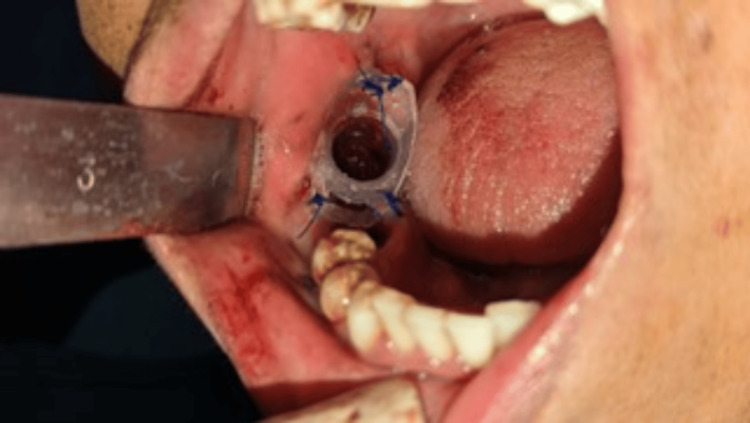
Placement of the marsupialization catheter

Following informed consent, a staged surgical approach was undertaken. Initially, decompression of the cyst was performed using marsupialization, which was maintained for three months. A marsupialization catheter was inserted into the bony cavity, secured with sutures, and designed to be small enough to avoid interference with daily mastication. This facilitated easy daily cleaning of the cystic cavity by the patient or clinical staff (Figure 4).

**Figure 5:**
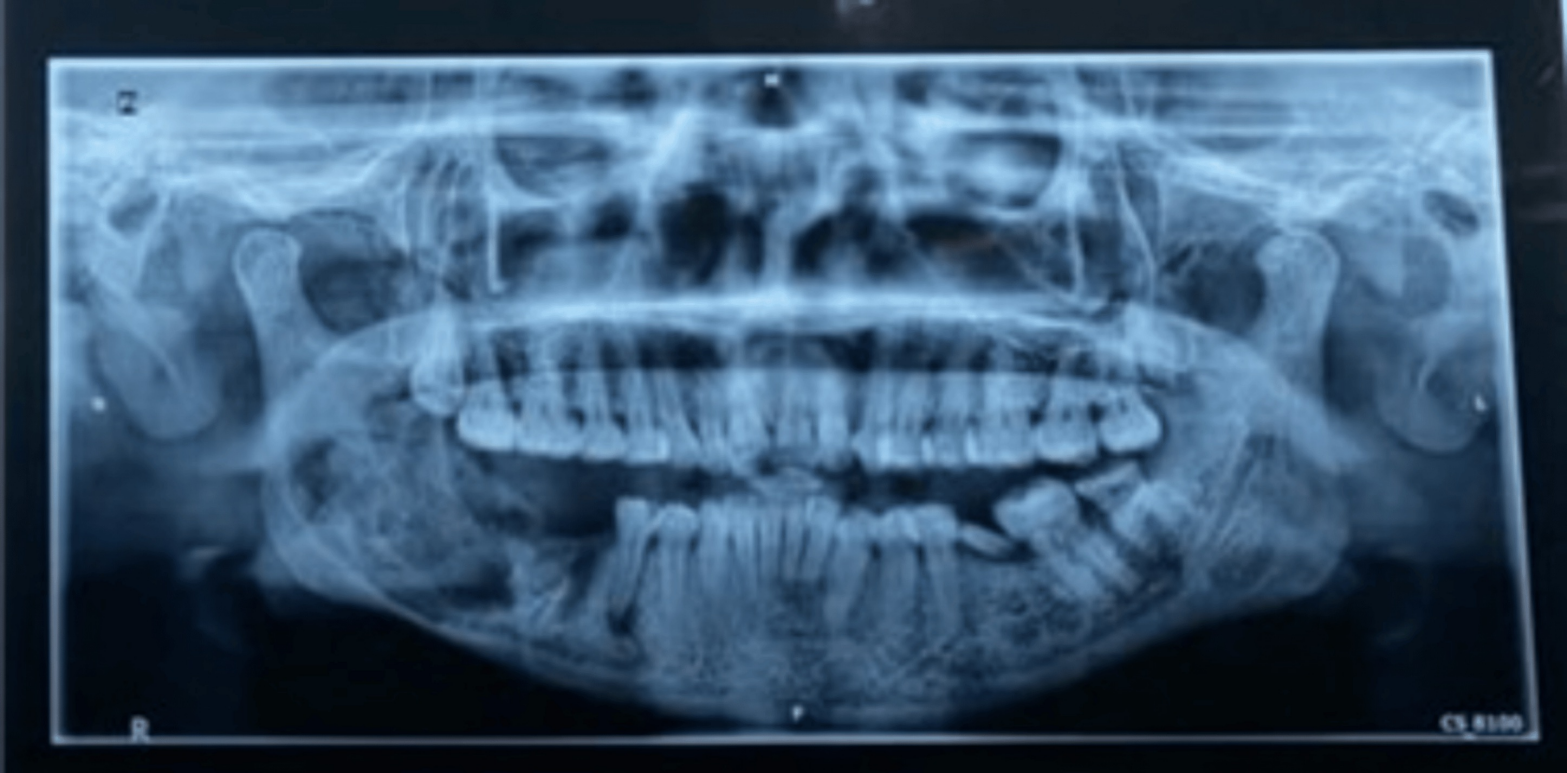
Orthopantomogram obtained after three months to assess the progress of decompression

An OPG was obtained after three months to assess the progress of decompression (Figure 5).

**Figure 6:**
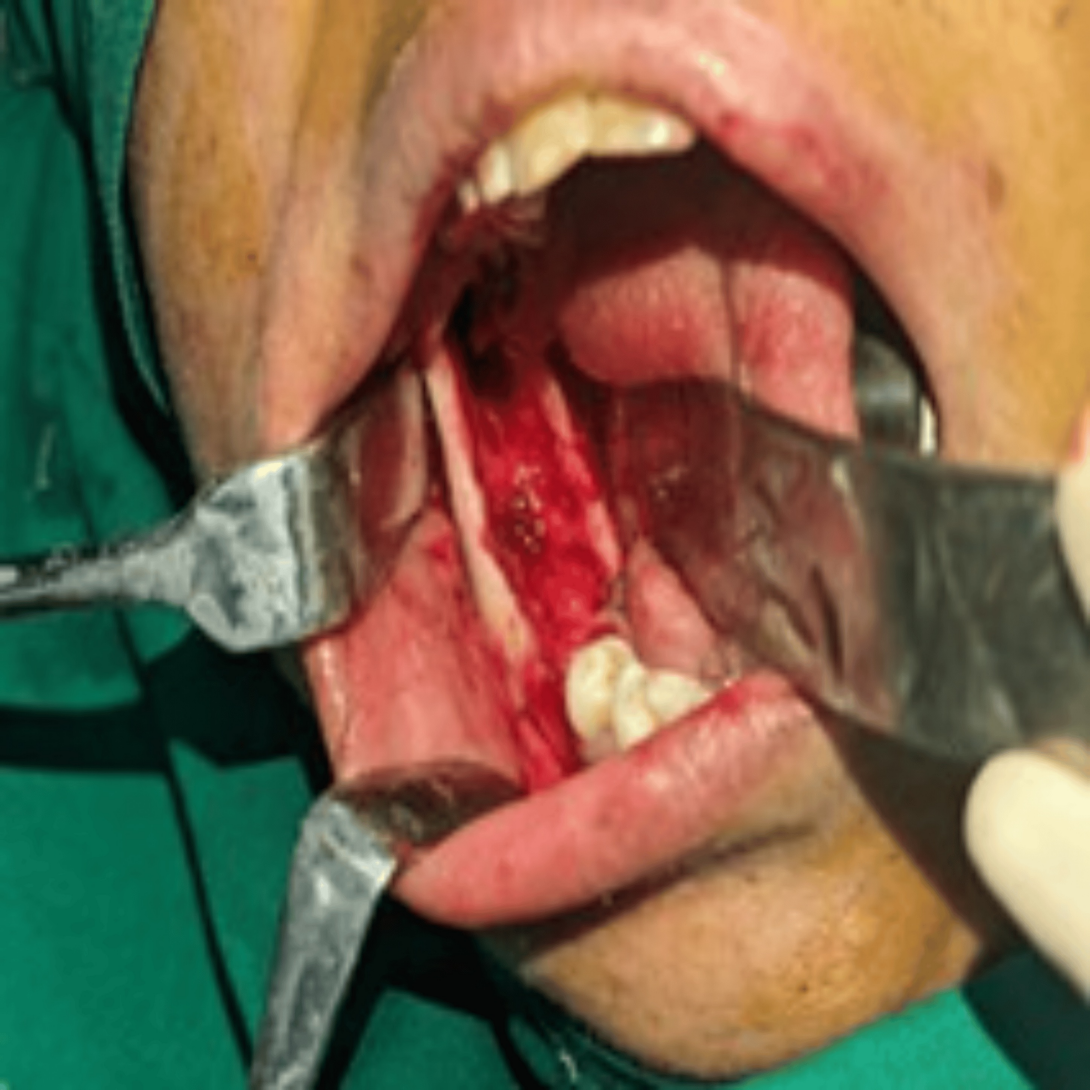
Intraoral image after enucleation of the cyst

**Figure 7:**
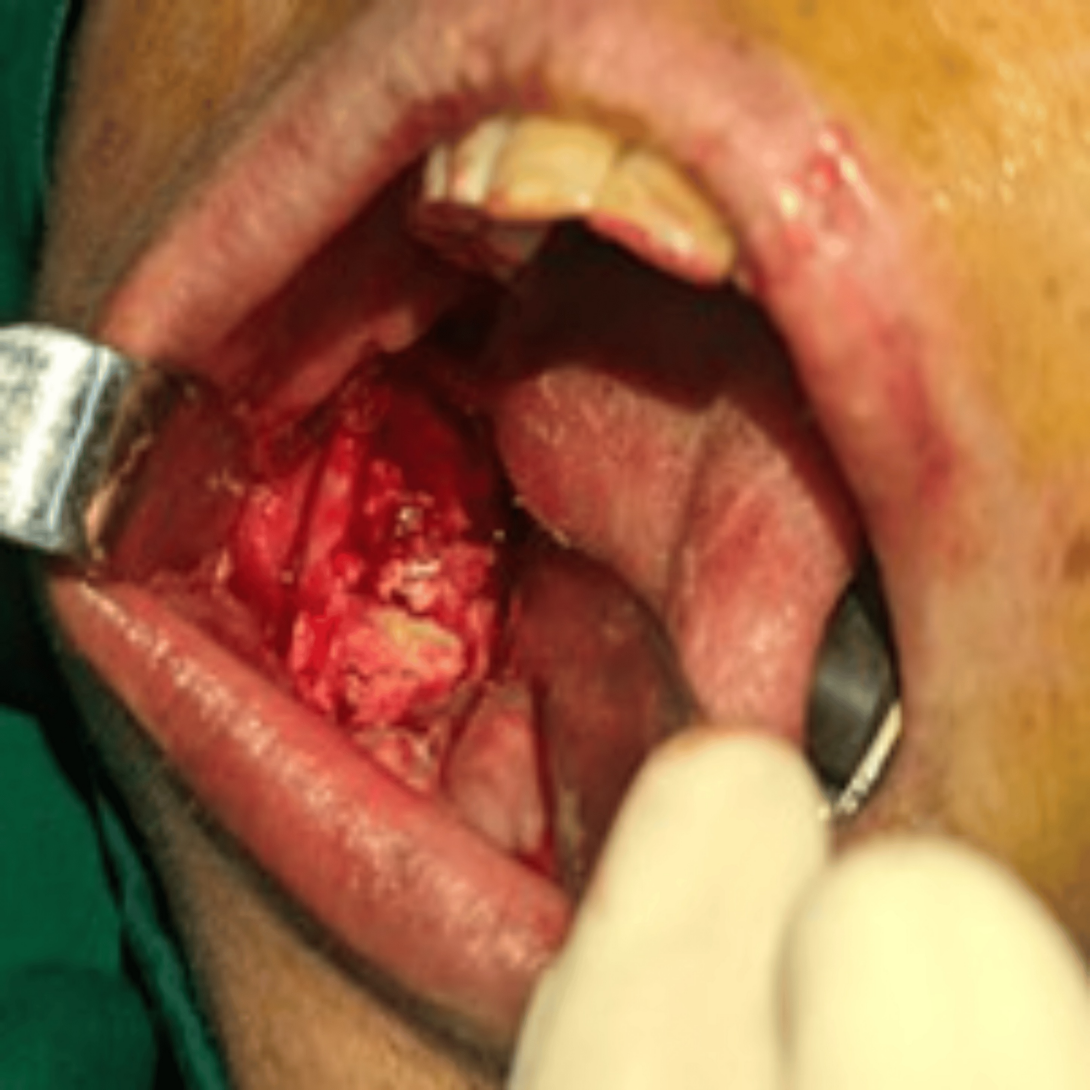
Intraoral image showing the placement of 5-FU-coated absorbable gelatin sponge into the surgical cavity 5-FU: 5-Fluorouracil

Subsequently, enucleation was performed under general anesthesia. A vestibular incision was made intraorally in the molar region, extending to the ascending ramus. The cyst was accessed through an existing perforation on the ramus, which was gradually extended using a round bur. Complete enucleation and debridement of the cystic cavity were performed, followed by copious irrigation with sterile normal saline. Topical 5% 5-FU was applied generously to all exposed bony walls using a cotton swab, and an absorbable gelatin sponge (Gelfoam) coated with 5% 5-FU was placed into the surgical cavity to obliterate the defect. The wound was then closed in a layer-wise manner (Figures 6, 7).

**Figure 8:**
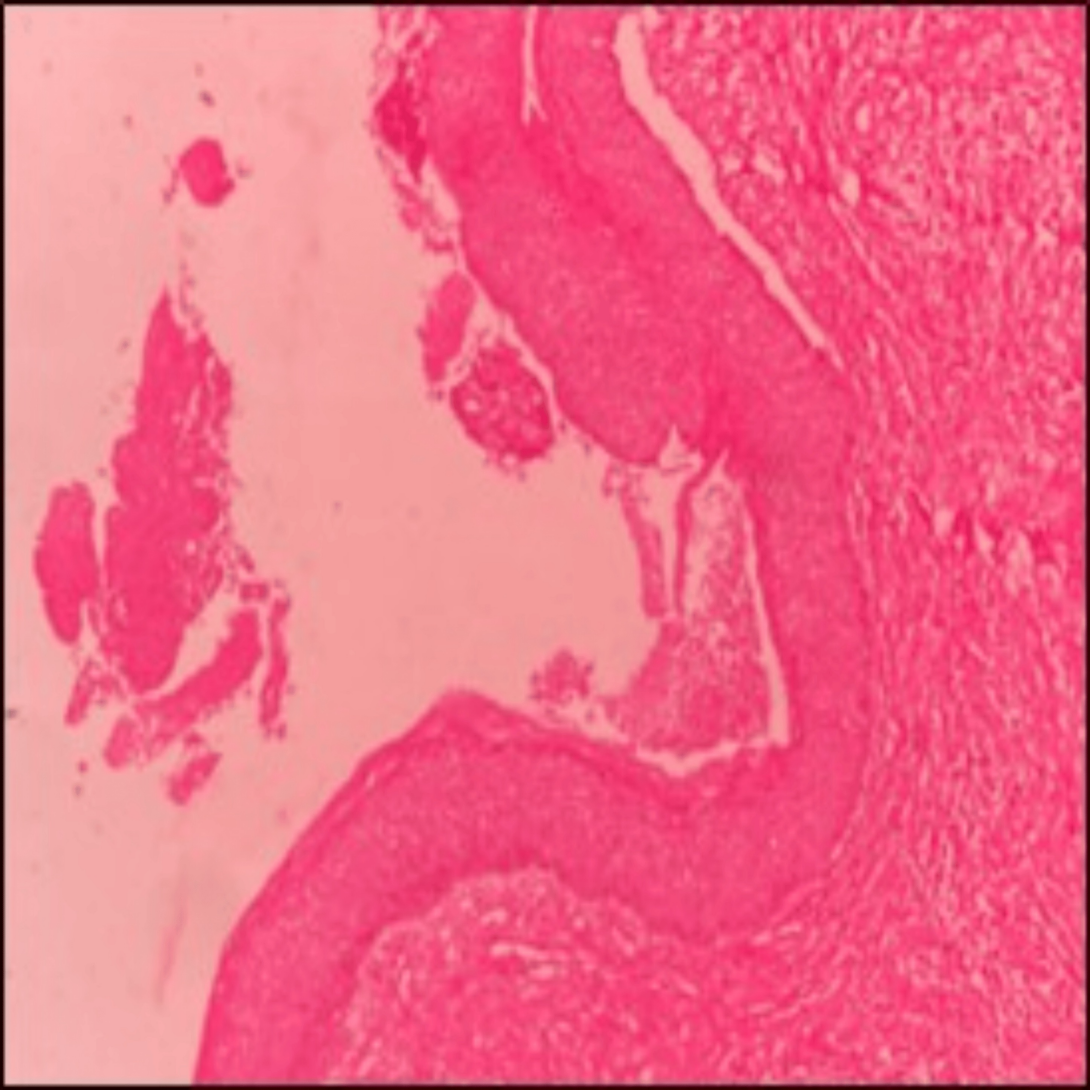
The cystic epithelium displayed variable thickness, predominantly 6–10 cell layers, with a corrugated parakeratinized surface and prominent basal cell hyperplasia

**Figure 9:**
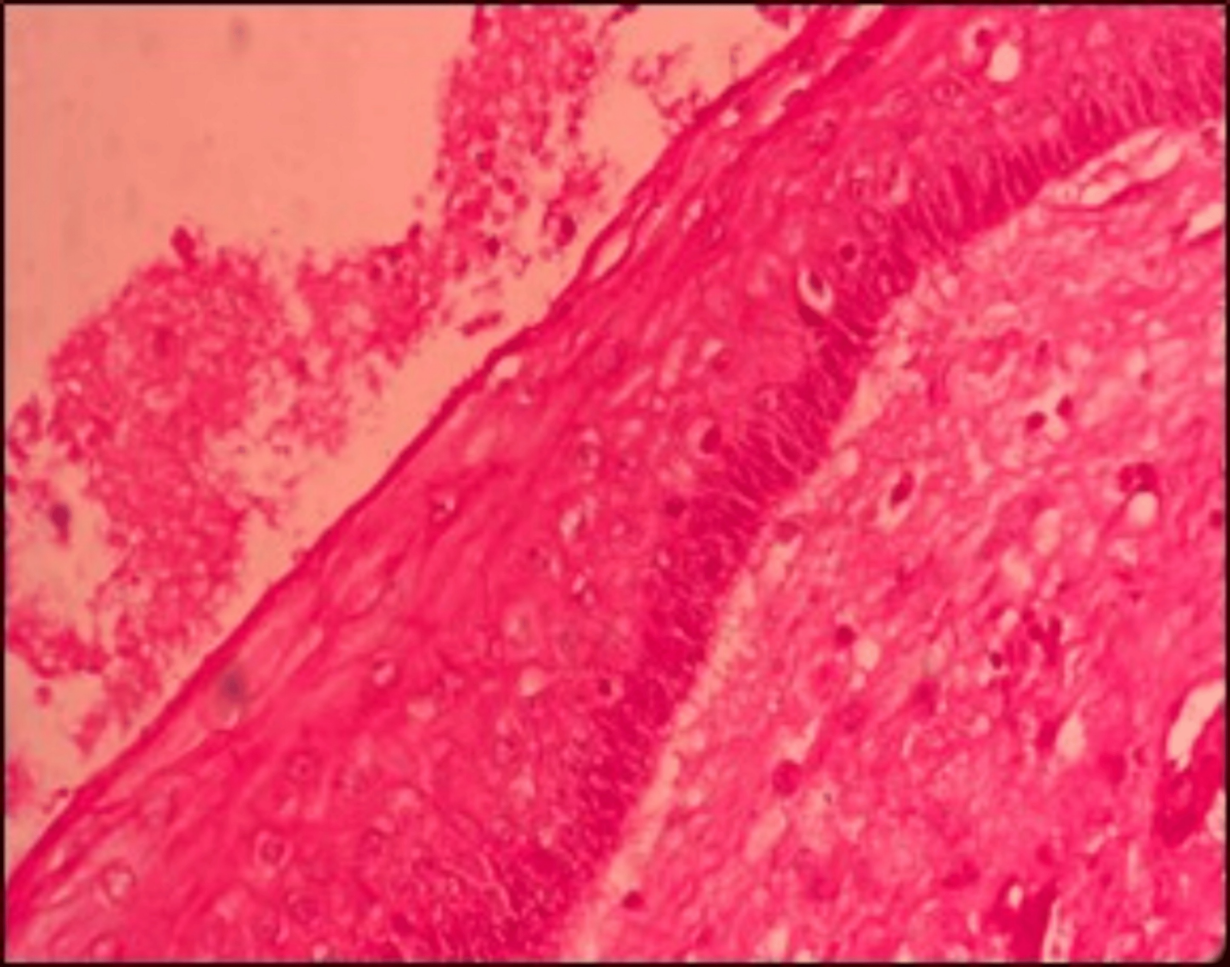
The basal cells were tall, columnar, and exhibited prominent palisading and polarized nuclei, with basal and suprabasal mitotic figures

Histopathological examination of the biopsy specimen confirmed the diagnosis of a parakeratinized OKC. The cystic epithelium displayed variable thickness, predominantly 6-10 cell layers (Figure 8), with a corrugated parakeratinized surface and prominent basal cell hyperplasia. The basal cells were tall, columnar and exhibited prominent palisading and polarized nuclei, with basal and suprabasal mitotic figures. Additional histological features included numerous epithelial infoldings, basal cell hamartomas, and prominent daughter cyst formation within the connective tissue capsule. The stroma consisted of delicate to densely arranged collagen fibers, blood vessels, and a mixed inflammatory infiltrate composed of lymphocytes, plasma cells, macrophages, and neutrophils. Areas of edematous stroma and reactive bone formation were observed in the deeper connective tissue. The cystic contents contained keratin, extravasated red blood cells, and inflammatory cells (Figure 9).

**Figure 10:**
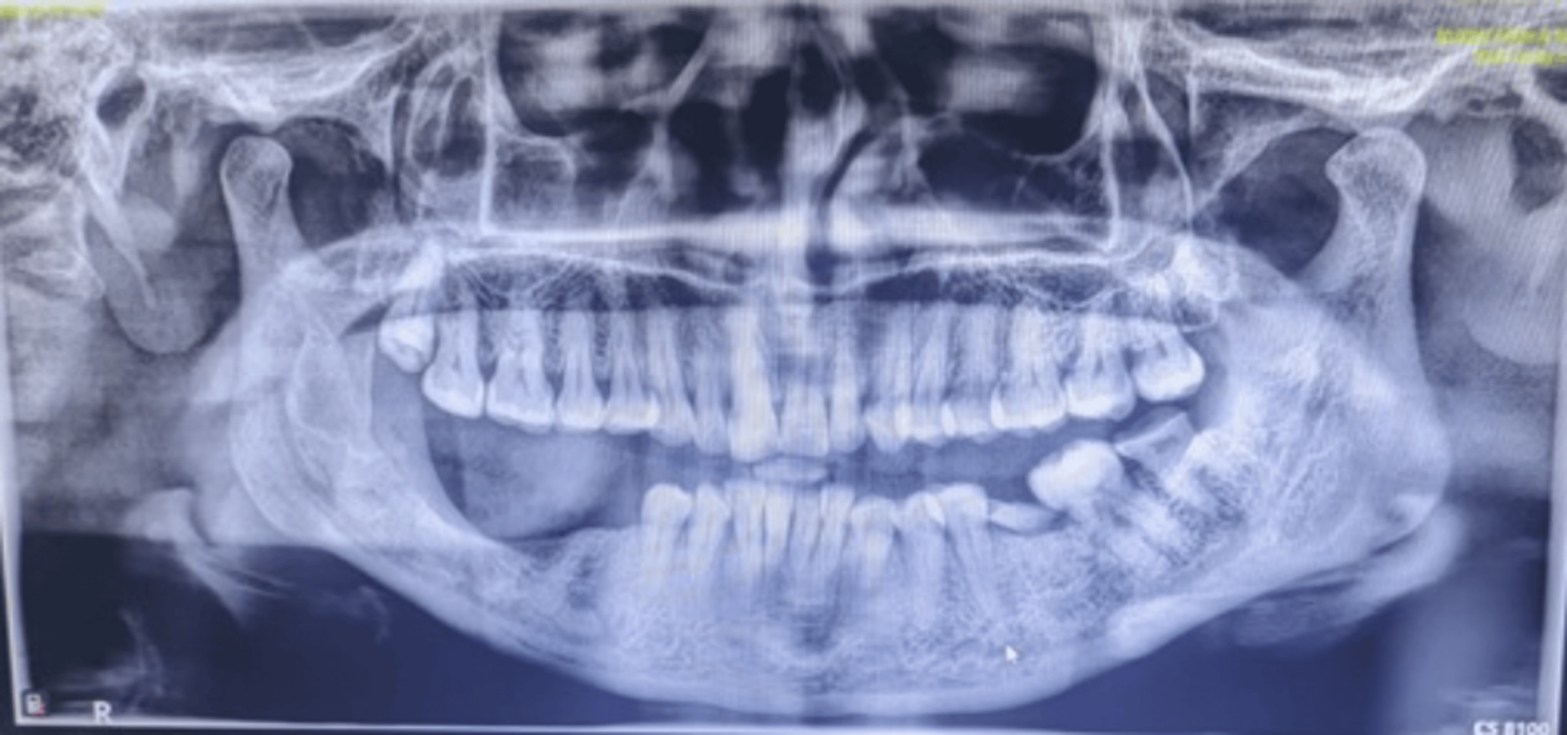
Orthopantomogram (OPG) assessment confirmed progressive bone regeneration and the absence of pathological changes

Radiographic follow-up at 12 months demonstrated new bone regeneration at the surgical site. The patient was monitored regularly at six, nine, and 12 months, with no signs of recurrence (Figure 10). OPG assessments confirmed progressive bone regeneration and the absence of pathological changes.

This case highlights the successful management of an OKC using a staged approach, incorporating marsupialization followed by enucleation and adjunctive application of 5-FU, contributing to favorable healing outcomes and reduced recurrence risk.

## Discussion

OKCs are unique and aggressive odontogenic cysts with a high recurrence rate, often behaving similarly to tumors. The primary reasons for recurrence include increased epithelial proliferative activity, basal layer budding, parakeratinization, supraepithelial and subepithelial splits, and the presence of epithelial remnants and daughter cysts. Various treatment modalities have been proposed for OKCs, including enucleation with or without adjunctive therapy, marsupialization followed by enucleation, and resection.

Marsupialization has been widely advocated as a conservative treatment option due to its ability to reduce lesion size, minimize morbidity, and preserve surrounding anatomical structures [2]. It reduces the aggressive behavior of OKCs by creating osmotic pressure on the surrounding tissues. The staged approach, initial marsupialization followed by enucleation, has been associated with lower recurrence rates compared to enucleation alone. Simple enucleation without adjuncts has been linked to recurrence rates ranging from 17% to 56% [2]. However, the addition of CS as a chemical fixative has significantly reduced recurrence rates to as low as 1.6% [2].

Dashow et al. [7] reported a 35% recurrence rate with enucleation and curettage combined with CS. MCS has been explored as a less cytotoxic alternative, though its effectiveness in reducing recurrence remains debatable [2]. A recent retrospective study suggested that MCS combined with peripheral ostectomy improves treatment outcomes [2].

The use of CS has been associated with delayed wound healing and potential neurotoxicity. In contrast, 5-FU not only reduces recurrence but also minimizes postoperative inferior alveolar nerve paresthesia. The therapeutic mechanism of 5-FU involves the inhibition of TS, leading to depletion of thymidine and preventing DNA replication, ultimately causing cell death. Moreover, 5-FU impacts multiple cellular pathways, including arachidonic acid metabolism and immune surveillance, which contribute to its antiproliferative effects [12]. The pharmacological response to 5-FU is influenced by three key enzymes: TS, thymidine phosphorylase (TP), and dihydropyrimidine dehydrogenase (DPD) [13]. While increased TP expression in inflamed OKC epithelium enhances 5-FU activation and its ability to eliminate residual epithelial cells, elevated DPD expression can reduce its efficacy by metabolizing it into inactive compounds [14]. Balamurgun et al. highlighted the efficacy of 5-FU as a promising treatment modality for OKCs [15]. These findings suggest that integrating 5-FU into treatment protocols could provide improved long-term outcomes with fewer complications. A study by Ledderhof et al. found that patients treated with MCS had an 18% recurrence rate over a follow-up period of 26.3 ± 1.8 months. However, no recurrence was observed in patients treated with 5-FU, indicating that 5-FU may be a more effective alternative to CS [16]. Further clinical studies are warranted to validate its efficacy and establish standardized treatment protocols for OKCs.

In the present case, a 35-year-old woman with a large OKC underwent a staged treatment approach. Initial marsupialization for three months facilitated decompression, reducing the lesion size and making subsequent enucleation less invasive. After enucleation, the application of 5% 5-FU directly to the bony cavity and its incorporation into absorbable gelatin sponges (Gelfoam) provided a chemical adjunct to prevent recurrence. This approach aligns with recent findings that advocate for 5-FU as a viable alternative to CS [14]. Histopathological examination confirmed the diagnosis of a parakeratinized OKC with daughter cyst formation, a factor known to contribute to high recurrence rates.

Radiographic follow-ups at six, nine, and 12 months revealed progressive bone regeneration without signs of recurrence, reinforcing the effectiveness of this treatment protocol. Compared to more aggressive surgical approaches like resection, this conservative yet effective management strategy preserved bone integrity while minimizing morbidity.

Thus, the integration of marsupialization, enucleation, and 5-FU application represents an effective, minimally invasive approach to OKC treatment, addressing both the need for recurrence prevention and improved postoperative healing. Further long-term studies are necessary to validate the superiority of 5-FU over conventional chemical adjuncts in OKC management.

## Conclusions

The management of OKCs remains a challenge due to their high recurrence rates and aggressive biological behavior. The present case highlights the efficacy of a staged treatment approach combining initial marsupialization, followed by enucleation and adjunctive application of 5% 5-FU. This protocol facilitated lesion size reduction, minimized surgical morbidity, and promoted favorable bone regeneration while effectively reducing recurrence risk.

Compared to conventional treatments such as enucleation with CS, 5-FU demonstrated potential advantages, including reduced neurotoxicity and improved postoperative healing. Radiographic follow-ups confirmed progressive bone formation without evidence of recurrence, supporting its viability as an alternative adjunctive therapy. Thus, the combination of marsupialization, enucleation, and 5-FU application presents a promising, minimally invasive approach to managing OKCs. Further long-term clinical studies are warranted to establish 5-FU’s superiority over other adjunctive agents in preventing recurrence and optimizing patient outcomes.
